# SymCART: a symbiotic cognitive-affective reinforcement transformer for optimizing educational interventions

**DOI:** 10.3389/fpsyg.2026.1784203

**Published:** 2026-07-02

**Authors:** Dan Liu, Feifei Li, Di Lu, Wenhua Yu, Runkai Jiao

**Affiliations:** 1 School of Business Management, Jilin University of Finance and Economics, Changchun, Jilin, China; 2 College of Education, Wenzhou University, Wenzhou, Zhejiang, China; 3 School of Medical Humanities, China Medical University, Shenyang, Liaoning, China; 4 School of Business Management, Jilin University of Finance and Economics, Changchun, Jilin, China; 5 School of Psychology, Northeast Normal University, Changchun, Jilin, China

**Keywords:** cognitive modeling, educational intervention, emotion analysis, multi-modal fusion, personalized education, reinforcement learning

## Abstract

Emotion and cognition analysis in educational interventions is crucial for enhancing personalized learning outcomes. However, existing models often encounter challenges in multimodal data integration and adaptive strategy optimization. To address these challenges, we propose the SymCART model, an intelligent educational intervention framework that integrates multimodal data fusion with deep reinforcement learning-based strategy optimization. SymCART dynamically adjusts teaching strategies through the collaborative operation of a multimodal perception encoder, dynamic cognitive-affective graph inference engine, and adaptive teaching strategy optimizer, thereby improving student learning outcomes. Experimental results demonstrate that SymCART achieves higher predictive accuracy and more effective strategy recommendations compared to traditional models, with statistically significant improvements in AUC, RMSE, weighted F1 for predictive tasks, and nDCG and ADR for policy/recommendation tasks across the IMPROVE, student learning behavior, and additional validation datasets. Ablation studies further confirm the essential contribution of each module, particularly regarding multimodal fusion and strategy optimization. The SymCART model provides robust support for personalized educational interventions and exhibits broad applicability for emotion and cognition analysis as well as adaptive learning strategies.

## Highlights

- Proposed SymCART model combines multi-modal data fusion and reinforcement learning for personalized educational interventions.
- Dynamic cognitive-affective graph reasoning enhances the model's ability to adjust teaching strategies based on both cognitive and emotional states of students.
- Superior performance demonstrated on IMPROVE and Student Learning Behavior datasets, outperforming existing state-of-the-art models.
- Multi-modal data fusion and adaptive teaching strategy optimization are crucial for enhancing student learning outcomes in real-time education environments.

## 1 Introduction

With the continuous advancement of artificial intelligence (AI) technologies, the education sector has increasingly integrated AI to enhance learning outcomes, teaching efficiency, and educational quality (Wang and Jiang, 2025; Zhou et al., 2025). Particularly in the intricate interplay between cognition and emotion, AI provides the capability to gain deeper insights into students' learning processes, analyze real-time cognitive and emotional states, and dynamically adjust teaching strategies based on these insights (Yang et al., 2026). Cognition and emotion, as two fundamental dimensions of human behavior, interact continuously during the learning process, profoundly influencing attention, memory, problem-solving, and overall learning effectiveness (Zhang et al., 2025). Despite the growing adoption of AI in education, most existing intervention methods primarily focus on a single dimension–either cognition or emotion–while largely neglecting their symbiotic and mutually reinforcing relationships. Furthermore, multimodal learning data, including physiological signals, behavioral observations, and engagement metrics, present challenges in data integration and temporal modeling. Against this backdrop, developing innovative AI-driven approaches that jointly consider cognition and emotion to optimize educational interventions has become a pressing and important issue in contemporary educational research (Yang, 2025). Addressing this problem requires models capable of handling high-dimensional multimodal data, capturing temporal dependencies, and adapting strategies dynamically to individual learners (Jian et al., 2025; Cui et al., 2025).

This paper proposes symbiotic cognitive-affective reinforcement transformer (SymCART), an intelligent framework designed to enhance educational interventions by simultaneously optimizing the interaction between cognition and emotion (Ning et al., 2026). SymCART leverages the Transformer architecture as the core framework, enabling hierarchical feature extraction, multi-level abstraction, and effective time-series modeling, which makes it particularly suitable for handling heterogeneous, multimodal learning data collected from students. Moreover, the model incorporates graph neural networks (GNNs) to capture relational and structural dependencies among cognitive and emotional states, and deep reinforcement learning (DRL) to dynamically optimize personalized teaching strategies. In complex educational environments, SymCART can finely adjust instructional actions based on real-time updates of students' emotional and cognitive states, thus effectively guiding the learning process. By jointly modeling cognition-emotion interactions and incorporating adaptive reinforcement learning, SymCART facilitates the development of individualized educational strategies, maximizes learning engagement, and improves learning outcomes across diverse student populations. The three main contributions of this paper are as follows:

- SymCART is proposed to address the limitation of traditional educational intervention methods that fail to simultaneously optimize cognitive and emotional states, achieving joint optimization of these two dimensions.

- An adaptive teaching strategy optimization method based on GNN and DRL is designed, addressing the challenge of dynamically adjusting teaching strategies in complex educational environments to maximize learning outcomes.

- The study provides empirical evidence to demonstrate the model's effectiveness, overcoming the limitations of existing educational interventions that cannot adequately assess and regulate the interaction between students' cognition and emotions.

The structure of this paper is as follows: The Section 1 introduces the background and motivation of the study. The Section 2 reviews the existing work related to this research and analyzes the shortcomings of current methods. The Section 3 provides a detailed description of the proposed SymCART model, including its architecture, key technologies, and implementation approach. The Section 4 presents the experimental design and result analysis, comparing the performance of different methods and conducting ablation experiments. Finally, the Section 5 summarizes the contributions of this paper and suggests potential directions for future research.

## 2 Related work

### 2.1 Approaches in cognitive and affective education

In the field of cognitive and emotional education, various methods have been developed to optimize teaching interventions. CNNs have been widely applied to process student behavioral data, extracting feature information to assess cognitive states and classify them through supervised learning models, thereby adjusting the teaching pace (Razzaq and Shah, 2025). In addition, CNN-based behavioral data analysis models can infer cognitive states by monitoring students' behavioral patterns and adapt teaching strategies accordingly (Bashir and Umar, 2025). While CNNs excel at capturing spatial and local feature patterns, they are limited in modeling long-term temporal dependencies, which can reduce performance in extended learning sequences. Decision Trees are commonly used for recommending personalized learning paths, automatically deriving the most suitable path for individual needs based on students' historical learning data (Chen et al., 2025). Although interpretable and efficient, Decision Trees can overfit to historical data and may struggle with complex, nonlinear relationships. LSTM networks, due to their powerful time-series processing capabilities, are frequently used to analyze students' emotional fluctuations and learning progress, predicting future emotional changes and making corresponding teaching adjustments (Adefemi and Mutanga, 2025; Alatrash et al., 2024). Despite their strengths, LSTMs are prone to vanishing gradients and may require extensive data to capture complex multimodal dependencies. Clustering Algorithms, especially K-means and DBSCAN, are used for classifying student groups and feature extraction, analyzing cognitive and emotional patterns within student groups to guide educators (Lu et al., 2025; Rahma and Ulfah, 2025). However, these unsupervised methods may be sensitive to noise and the selection of hyperparameters, limiting their generalizability. Principal component analysis (PCA) reduces the dimensionality of student data and helps discover correlations between emotion and cognition (Syed Mustapha, 2023), yet it is a linear method that cannot capture nonlinear interactions. Graph convolutional networks (GCNs) construct relational graphs among students to capture dynamic changes in their emotions and cognition (Sönmez and Varol, 2020), but they rely on carefully designed graph structures and may not generalize well across different datasets. Additionally, SVMs are used for emotion classification by analyzing student behavior and physiological signals (Shixin et al., 2024), though they require feature engineering and may not scale efficiently with large datasets. Autoencoder methods extract low-dimensional features from high-dimensional multimodal data to optimize emotion-cognition fusion, improving intervention effectiveness, yet their performance heavily depends on network architecture and training stability. Although these methods have achieved certain results, most focus only on optimizing a single dimension of cognition or emotion, lacking joint modeling of their symbiotic relationship (Shukla et al., 2024).

In contrast to these methods, the SymCART model not only captures the complex relationship between students' emotional fluctuations and cognitive progress but also adapts intervention strategies in response to the dynamic nature of the teaching environment. Through this multidimensional optimization, SymCART provides students with personalized learning experiences, significantly enhancing the intelligence of educational interventions.

### 2.2 Approaches for optimizing educational interventions

Many methods have been proposed to improve learning outcomes in optimizing educational interventions (Lin et al., 2025). For example, MLPs are commonly used in learning behavior analysis, where their hierarchical structure is employed to model students' learning patterns and predict their learning progress (Tang et al., 2024). While MLPs efficiently process student learning data, their drawback lies in the inability to effectively capture dynamic changes in time-series data. Moreover, when dealing with complex, multidimensional data, the performance of MLPs can be limited (Pang and Wei, 2025). GANs are widely used to simulate the interaction between students' emotions and cognition, generating virtual data that aligns with student behavior, thereby optimizing personalized learning paths. However, instability during GANs' training process and the quality of generated data pose significant challenges, especially in the case of highly diverse and complex educational data (Adefemi et al., 2025). RNNs perform well in handling time-series data, particularly in emotion prediction and learning behavior analysis. Despite their ability to capture dependencies in time-series data, RNNs struggle with issues such as vanishing or exploding gradients in long sequences, and their computational efficiency can be low (Emima and Amalarethinam, 2025). Deep belief networks (DBNs) are used for unsupervised learning of students' historical learning data, identifying latent patterns in their cognition and emotion. While DBNs excel in data dimensionality reduction and feature extraction, their modeling ability for high-dimensional and complex data is limited, and their training process is relatively complex (Fahid et al., 2024). RL is applied to optimize personalized learning strategies, adjusting teaching content adaptively through a reward mechanism to improve learning outcomes. While RL can dynamically adjust strategies, it requires large amounts of training data and time, and the design of appropriate reward functions remains a challenge in practical applications (Liu and Zhu, 2025; Wang et al., 2025).

In contrast to these methods, the SymCART model proposed in this paper achieves multidimensional joint optimization of cognition and emotion by integrating Transformer, GNN, and DRL. Unlike traditional methods that focus on optimizing a single dimension, SymCART dynamically adjusts teaching strategies, fully considering the symbiotic relationship between students' emotions and cognition, thereby providing a more intelligent and personalized solution for educational interventions.

## 3 Model

### 3.1 Overview of our model

**Figure 1:**
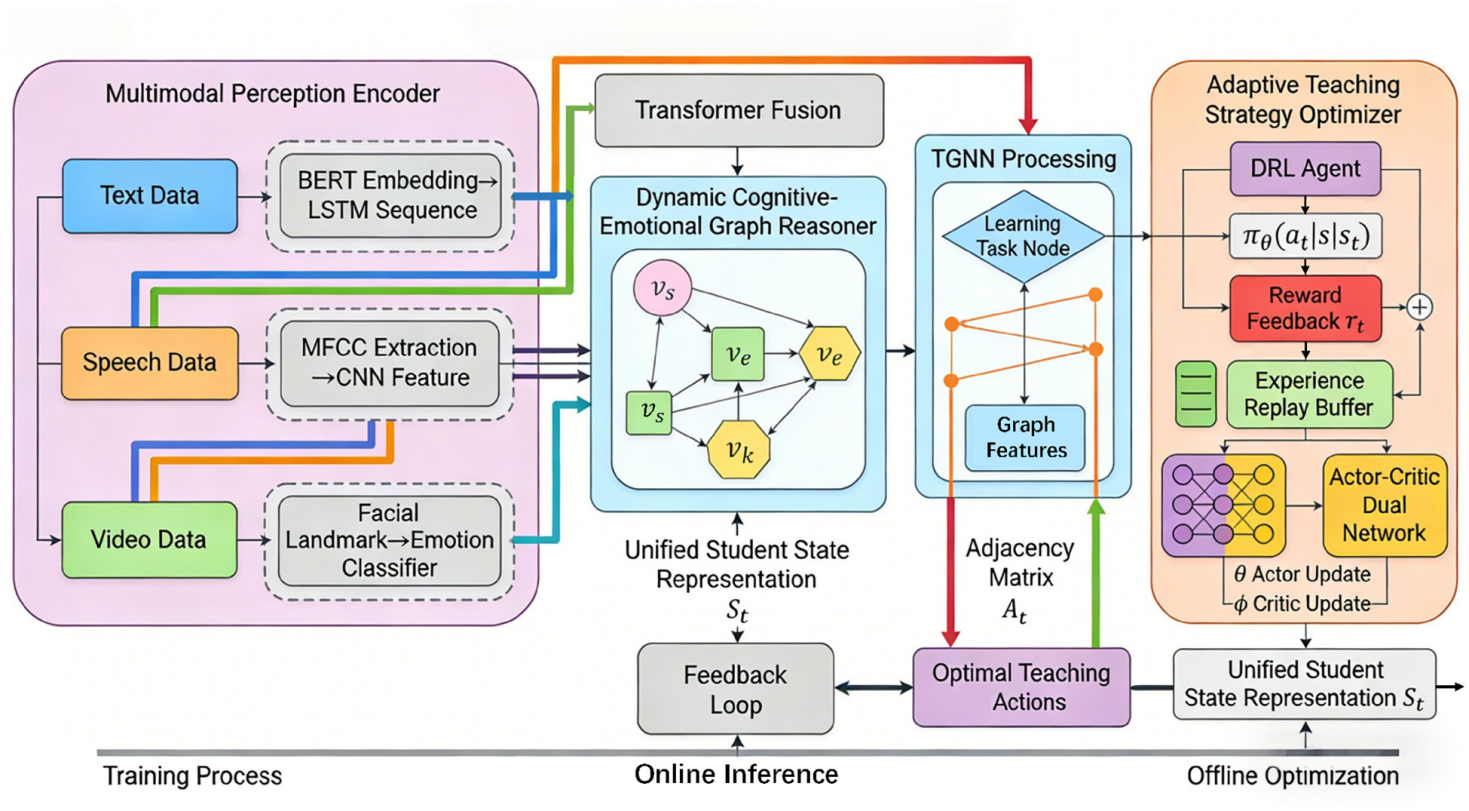
Overall architecture of the SymCART model.

The SymCART model proposed in this paper optimizes educational intervention strategies through the fusion and processing of multimodal data. By utilizing the collaborative efforts of multiple modules, the model dynamically captures changes in students' emotional and cognitive states and adjusts the teaching content based on real-time feedback from students, thereby achieving personalized educational interventions. The overall framework of the model includes several functional modules, where the raw data is fused and processed by the multimodal perception encoder, then passed to the dynamic cognitive-affective graph inference engine for state modeling and reasoning, and finally flows into the adaptive teaching strategy optimizer for personalized decision-making and optimization. Each module plays a distinct role within the overall architecture, and their collaboration ensures that the model can adapt to complex educational environments and optimize teaching outcomes. The design of the model structure is illustrated in Figure 1, showcasing the functions of each module and the data flow between them.

The multimodal perception encoder is responsible for extracting key information from students' multimodal data, including text, speech, and video. Leveraging the powerful capabilities of the Transformer architecture, the model effectively fuses these heterogeneous data types into a unified student state representation, providing comprehensive emotional and cognitive information. This module not only helps the model identify students' emotional fluctuations but also captures their learning behaviors and cognitive levels, providing accurate input data for subsequent modules (Du et al., 2025). By analyzing students' state changes in real time, the encoder lays the necessary perceptual foundation for the entire model. The dynamic cognitive-affective graph inference engine builds a relational graph between student states based on a temporal graph neural network, dynamically adjusting the flow of emotional and cognitive information. This module analyzes the historical behavioral relationships between students, identifies potential emotional and cognitive patterns, and outputs graph features that aid decision-making. This process enables the model to infer possible future emotional and cognitive changes based on students' historical behaviors, further enhancing the specificity and adaptability of the educational intervention. The adaptive teaching strategy optimizer combines deep reinforcement learning techniques to dynamically adjust teaching strategies based on students' emotional and cognitive states (Wang et al., 2026). Through reinforcement learning, the model can adapt learning tasks according to each student's learning progress and feedback, optimizing personalized educational interventions. This module not only considers students' immediate emotional responses but also takes their cognitive development as an optimization target, resulting in more accurate learning path recommendations. Through long-term strategy optimization, the model can achieve continuous improvement in students' learning outcomes.

Overall, the SymCART model achieves a high level of coordination between its modules, with the output of each module serving as the foundation for the optimization decisions of the next module. Through effective fusion of multimodal data and dynamic adjustments of deep learning algorithms, the model is able to implement precise personalized interventions in complex educational environments. This joint optimization mechanism not only enhances the flexibility of teaching strategies but also increases the intelligence of educational interventions.

### 3.2 Multimodal perception encoder for student state representation

**Figure 2:**
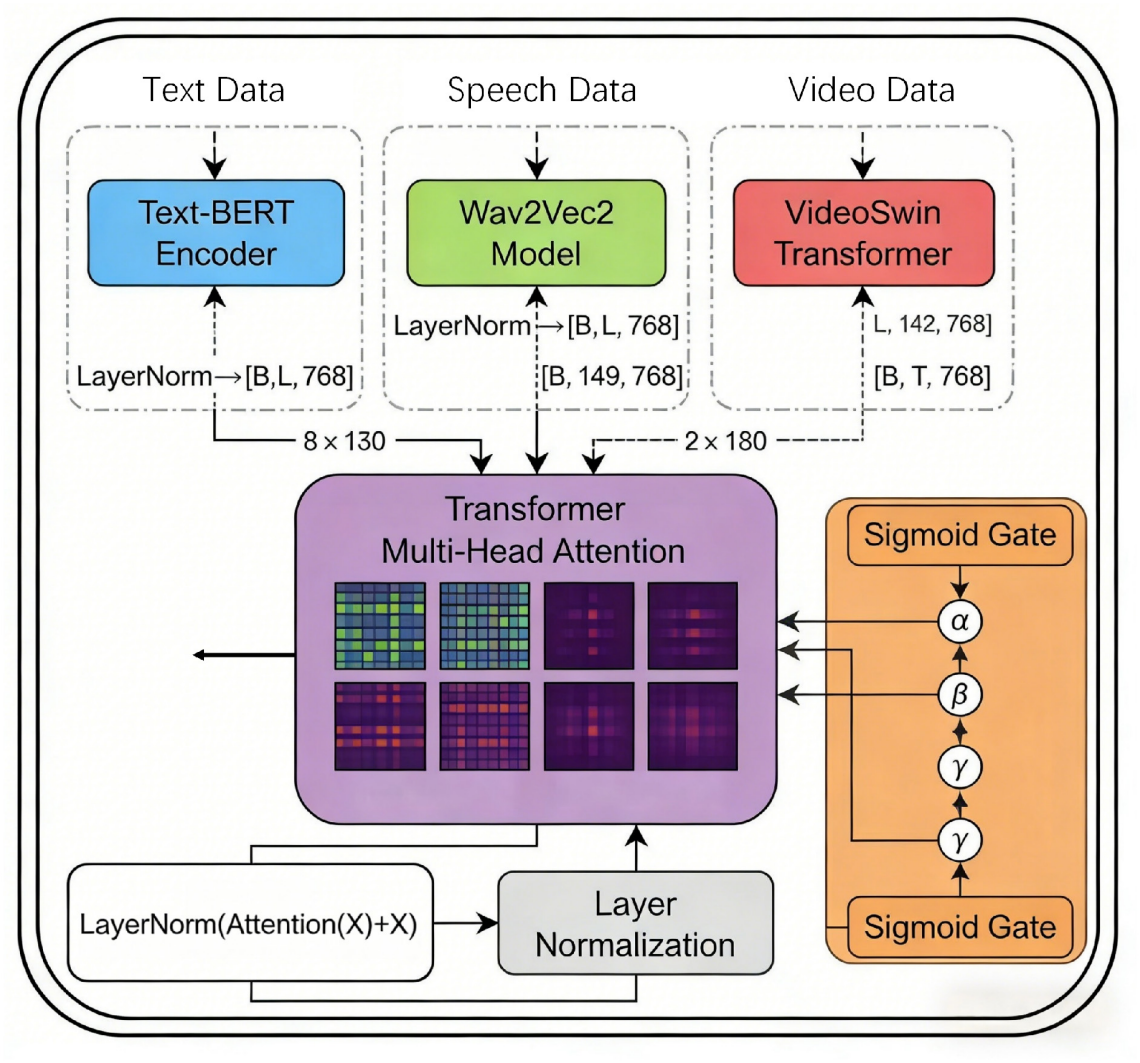
Architecture of the multimodal perception encoder.

The multimodal perception encoder is a core component of the SymCART model, responsible for extracting key information from students' multimodal data, including text, speech, and video. In this module, the raw multimodal input data are first processed through pre-trained encoders for feature extraction, generating initial feature vectors. The self-attention mechanism of the Transformer then performs weighted fusion of these features, creating a unified student state representation, which provides multimodal emotional and cognitive information for subsequent modules. Figure 2 illustrates the specific structure and data flow of this module, clearly reflecting the processing and fusion process of each modality's features.

This module integrates heterogeneous data from text, audio, and video into a unified student state representation. Text data is processed through the Text-BERT encoder, audio data through the Wav2Vec2 model, and video data through the VideoSwin model. Using these pre-trained models, each modality's data is transformed into specific feature vectors, which are then weighted according to their impact on emotion and cognition during the learning process.

The expression $H_{t}=f_{\text{Text}-\text{BERT}}\left(X_{t}\right)\in {\mathbb{R}}^{L_{t}\times d_{t}}$ represents the feature vector *H*_*t*_ extracted from the text data *X*_*t*_, where *L*_*t*_ is the sequence length and *d*_*t*_ is the feature dimension. Similarly, the audio and video data are processed through Wav2Vec2 and VideoSwin to extract features, yielding $H_{a}=f_{\text{Wav}2\text{Vec}2}\left(X_{a}\right)\in {\mathbb{R}}^{L_{a}\times d_{a}}$ and $H_{v}=f_{\text{VideoSwin}}\left(X_{v}\right)\in {\mathbb{R}}^{L_{v}\times d_{v}}$, where *L*_*a*_ and *L*_*v*_ represent the sequence lengths for audio and video, and *d*_*a*_ and *d*_*v*_ are their respective feature dimensions. Using these pre-trained models, the features for each modality are independently extracted and represented as high-dimensional feature vectors, providing multimodal information for subsequent modules.

To unify the features of all modalities, we perform a linear projection to map them into the same feature space d. *Z*_*t*_, *Z*_*a*_, and *Z*_*v*_ represent the aligned features of the text, audio, and video modalities, respectively. Through this approach, the data from all modalities are transformed into a unified feature space, ensuring that effective fusion between modalities is possible. Calculate as shown in Equation 1:


$$\begin{array}{l} Z_{i}=\text{Linea}{\text{r}}_{i}\left(H_{i}\right)\in {\mathbb{R}}^{L\times d},i\in \left\{t,a,v\right\} \end{array}$$ (1)

The fused features will undergo deep fusion through a cross-modal attention mechanism. The text modality features *Z*_*t*_ serve as the query, and attention weights are computed by concatenating the audio and video features, Concat(*Z*_*a*_, *Z*_*v*_). *Q*, *K*, and *V* represent the query, key, and value, respectively, denoting the features from different modalities. Through this mechanism, the text modality can "attend" to the relevant information from the audio and video modalities, enabling deep fusion of information in the modeling of emotion and cognition. The fused feature *F*_*t*_ for the text modality is obtained in a similar manner, and analogous fusion operations are applied to the audio and video modalities, generating the *F*_*a*_ and *F*_*v*_ features, respectively. Calculate as shown in Equations 2 and 3:


$$\begin{array}{l} \text{CrossAttn}\left(Q,K,V\right)=\text{softmax}\left(\frac{QK^{T}}{\sqrt{d}}\right)V \end{array}$$ (2)

$$\begin{array}{l} F_{t}=\text{CrossAttn}\left(Q=Z_{t},K=\text{Concat}\left(Z_{a},Z_{v}\right),V=\text{Concat}\left(Z_{a},Z_{v}\right)\right) \end{array}$$ (3)

For each given time window, the fused features from all modalities are aggregated into a unified student state representation *S*_*t*_. This aggregation process is performed using mean pooling (MeanPooling). *W*_*t*_, *W*_*a*_, and *W*_*v*_ are learnable projection matrices, and *b* is the bias term. The aggregation results in a fixed-length vector *S*_*t*_, which serves as the output of this module and is passed to the downstream dynamic cognitive-affective graph inference engine module for further processing. Through this series of operations, the encoder is able to effectively capture the multimodal emotional and cognitive states of the student, providing precise informational support for subsequent educational interventions. Calculate as shown in Equations 4 and 5:


$$\begin{array}{l} F_{\text{fused}}=\text{LayerNorm}\left(W_{t}F_{t}+W_{a}F_{a}+W_{v}F_{v}+b\right) \end{array}$$ (4)

$$\begin{array}{l} S_{t}=\text{MeanPooling}\left(F_{\text{fused}}\right)\in {\mathbb{R}}^{d} \end{array}$$ (5)

The multimodal perception encoder plays a crucial role in the SymCART model. By deeply integrating and extracting features from various data modalities, it provides strong support for the subsequent cognitive and emotional reasoning modules. This module not only effectively captures students' emotional fluctuations in different contexts but also accurately analyzes their cognitive states, ensuring that the model can adapt flexibly to various educational intervention scenarios. Through real-time perception and dynamic data flow, the model is able to offer comprehensive informational support for educational decision-making, thereby enhancing the personalization and intelligence of educational interventions.

### 3.3 Dynamic cognitive-affective graph inference for personalized educational intervention

**Figure 3:**
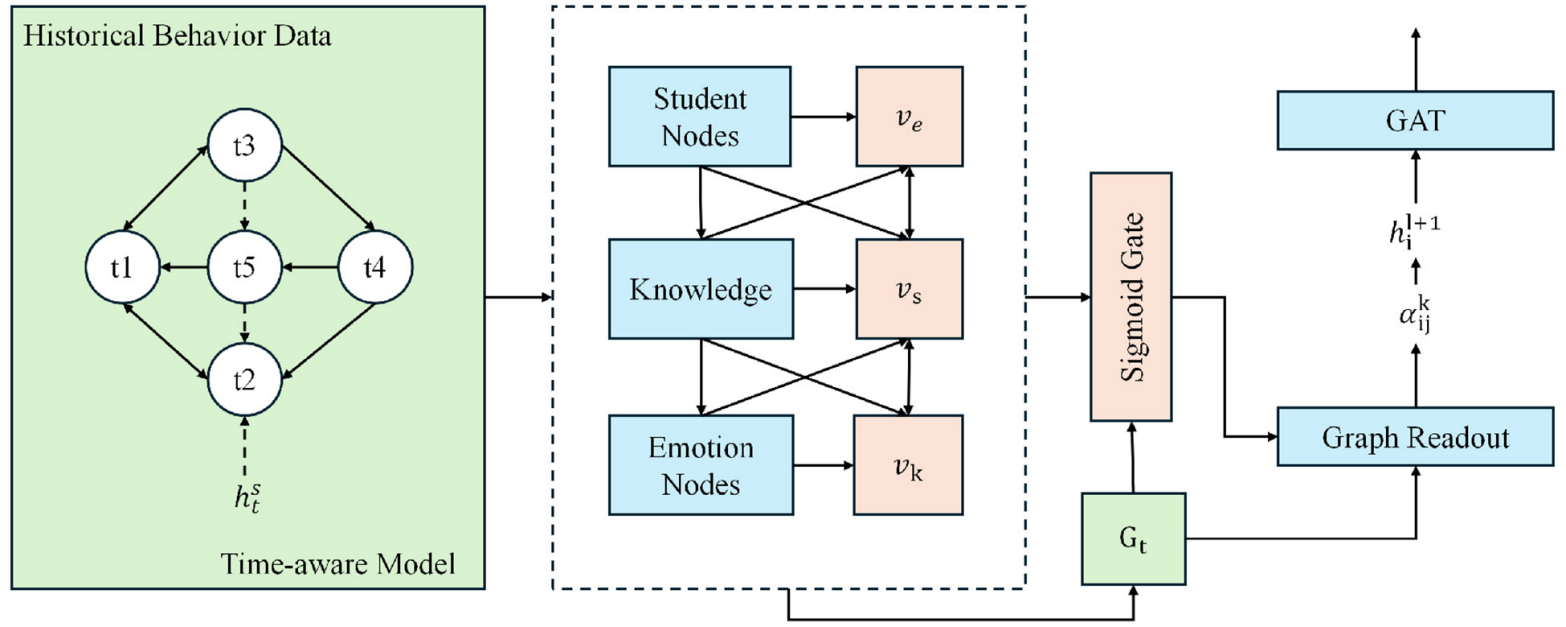
Architecture of the dynamic cognitive-affective graph inference module.

The dynamic cognitive-affective graph inference engine plays a pivotal role in the SymCART model, responsible for constructing and updating the emotional and cognitive relationship graph based on students' historical behaviors. This module dynamically adjusts the transmission of emotional and cognitive information at each time step. Each node in the graph represents a student's emotional or cognitive state, while the edges reflect the relationships between students or between students and learning tasks or knowledge points. By analyzing the historical behavioral relationships and emotional changes among students, the TGNN captures the dynamic evolution of students' emotions and cognition, enabling the model to infer potential future emotional fluctuations and cognitive changes. This provides more precise decision-making support for educational interventions. Figure 3 illustrates the structure and workflow of this module, showcasing how the graph neural network enables deep modeling and updating of emotional and cognitive information.

The dynamic cognitive-affective graph inference engine plays a crucial role in the SymCART model, responsible for dynamically updating the emotional and cognitive relationship graph based on students' historical behaviors. At each time step, the state vector *S*_*t*_ from the perception encoder is used to update the relevant node states, typically the student node $v_{t}^{s}$ and the most active emotional node $v_{t}^{s}$. This node embedding update is performed using a GRU to capture the temporal dependencies in the state evolution. $h_{t-1}^{s}$ represents the hidden state of the student node at the previous time step, while *W*_*s*_ and *b*_*s*_ are learnable parameters, and *S*_*t*_ is the state vector output from the perception encoder. Similarly, the embeddings of the most relevant emotional node *v*_*e*_ and knowledge point node *v*_*t*_ related to the current learning activity are updated, ensuring that the nodes in the graph can adjust according to the new emotional and cognitive information. Calculate as shown in Equation 6:


$$\begin{array}{l} h_{t}^{s}=\text{GRU}\left(h_{t-1}^{s},W_{s}S_{t}+b_{s}\right) \end{array}$$ (6)

The edges between nodes in the graph are also updated according to changes in the node states. The edge weights (or features) reflect the strength of the relationship between the student and the knowledge point. σ represents the Sigmoid function, and || denotes vector concatenation. *W*_*a*_ and *b*_*a*_ are learnable parameters, while $h_{t}^{s}$ and $h_{t}^{k}$ are the state vectors of the student node and knowledge point node, respectively. The weight $a_{t}^{e}$ can be interpreted as the “mastery” or “attention” of the student *v*_*s*_ on the knowledge point *v*_*k*_ at the current moment. This weight plays a crucial role in calculating the information transfer and aggregation between nodes. Calculate as shown in Equation 7:


$$\begin{array}{l} a_{t}^{e}=\sigma \left(W_{a}\left[h_{t}^{s}||h_{t}^{k}\right]+b_{a}\right) \end{array}$$ (7)

After updating the node and edge states, information propagates between nodes through a GCN. We adopt the message-passing mechanism of GAT, which allows nodes to aggregate information from their neighbors. $h_{i}^{l}$ represents the feature of node *i* after the *l*-th layer of graph convolution, and *N*(*i*) is the set of neighbors of node *i*. $\alpha_{ij}^{k}$ is the normalized attention coefficient calculated by the *k*-th attention head. This coefficient represents the importance of node *j* to node *i*, computed jointly based on node and edge features. *W*_*k*_ is the weight for the *k*-th attention head, and *K* is the number of attention heads. Calculate as shown in Equation 8:


$$\begin{array}{l} h_{i}^{l+1}={||}_{k=1}^{K}\sigma \left(\underset{j\in N\left(i\right)}{\sum }\alpha_{ij}^{k}W_{k}h_{j}^{l}\right) \end{array}$$ (8)

After *L* layers of propagation, we obtain the updated high-order representations of all nodes $h_{i}^{L}$. These representations are then aggregated into a global representation *G*_*t*_ of the entire graph through a graph readout function. Calculate as shown in Equation 9:


$$\begin{array}{l} G_{t}=\text{READOUT}\left(\left\{h_{i}^{L}|v_{i}\in V_{t}\right\}\right)=\frac{1}{\left|V_{t}\right|}\underset{v_{i}\in V_{t}}{\sum }h_{i}^{L} \end{array}$$ (9)

The READOUT function here uses a simple global mean pooling, but it can also be designed as a more complex attention-based pooling mechanism. In this way, the TGNN module integrates the features of each student node and its relational edges, generating a global, graph-level representation of emotional and cognitive states. This global representation serves as an important basis for the subsequent optimization of teaching strategies.

### 3.4 Adaptive teaching strategy optimization with deep reinforcement learning

**Figure 4:**
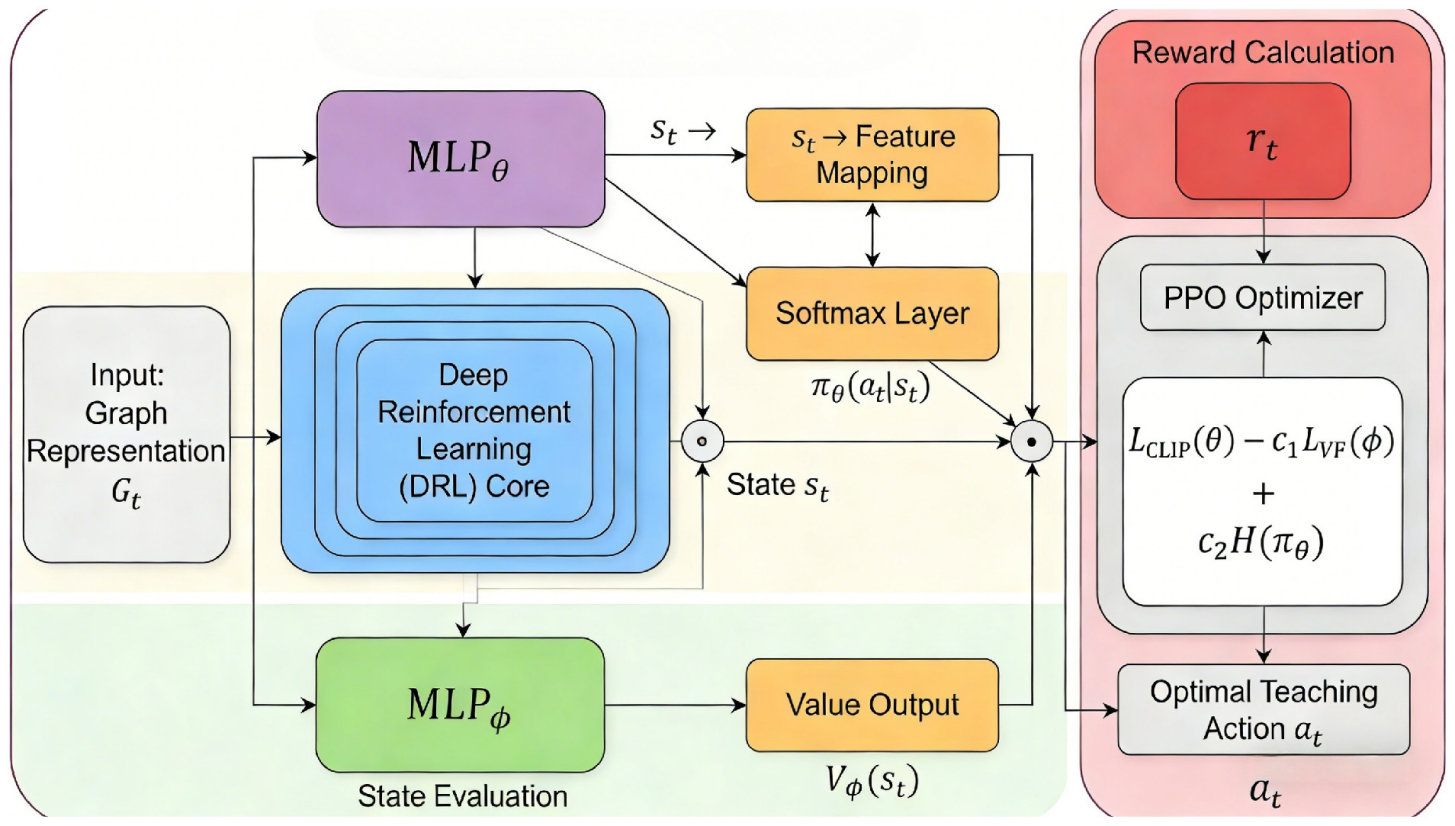
Structure of the adaptive teaching strategy optimization module.

The adaptive teaching strategy optimizer plays a core role in the SymCART model, responsible for dynamically adjusting the teaching content based on students' emotional and cognitive states. This module employs DRL techniques, using a policy network to select the optimal teaching intervention actions in real-time, thereby enhancing the effectiveness of personalized educational interventions. By integrating students' emotional and cognitive information, the module can automatically adjust the teaching content to align with students' learning needs and progress. Figure 4 illustrates the structure and data flow of this module, demonstrating how deep reinforcement learning algorithms are utilized to improve the precision and personalization of educational intervention strategies.

The adaptive teaching strategy optimizer is a key component of the SymCART model, responsible for dynamically adjusting teaching content based on students' cognitive and emotional states to optimize the effectiveness of personalized educational interventions. In the model, the policy network takes the dynamically generated graph features *G*_*t*_ as input and outputs action decisions for the current state. Specifically, the policy function π_θ_(*a*_*t*_|*s*_*t*_) guides educational decisions by calculating the probability distribution of actions given the current state. *W*_π_ and *b*_π_ are learnable parameters, and MLP MLP_θ_ is a multi-layer perceptron (MLP) network that processes the student *s*_*t*_ and outputs the action probability distribution. This policy network helps the agent select the optimal teaching action. Calculate as shown in Equation 10:


$$\begin{array}{l} \pi_{\theta }\left(a_{t}|s_{t}\right)=\text{softmax}\left(W_{\pi }\text{ML}{\text{P}}_{\theta }\left(s_{t}\right)+b_{\pi }\right) \end{array}$$ (10)

To evaluate the quality of states or state-action pairs, the model also needs to learn a value function to measure the expected cumulative reward that can be obtained by following the current policy in a given state. The state value function *V*_ϕ_(*s*_*t*_) and the action value function *Q*_ϕ_(*s*_*t*_, *a*_*t*_) are used to assess the student's cognitive and emotional states, respectively. *w*_*v*_ and *b*_*v*_ are learnable parameters, and MLP MLP_ϕ_(*s*_*t*_) is a MLP used for state mapping. This function measures the potential learning reward of the student in a given state and helps the policy network make better decisions. Calculate as shown in Equation 11:


$$\begin{array}{l} V_{\phi }\left(s_{t}\right)=w_{v}^{⊤}\text{ML}{\text{P}}_{\phi }\left(s_{t}\right)+b_{v} \end{array}$$ (11)

The ultimate goal of the agent is to maximize the expected cumulative discounted reward *J*(θ), where the reinforcement learning problem is formulated as a Markov decision process (MDP) (*S, A, P, R*, γ). Here, *S* represents the state space, capturing students' cognitive and emotional states at each teaching step and encoded as multimodal feature vectors including text, audio, video, and knowledge mastery information. The action space *A* is explicitly defined, enumerating all possible teaching/intervention actions, including action types (e.g., quiz assignments, instructional videos, feedback messages), granularity (topic-level vs. subtopic-level), and whether actions are discrete or continuous, along with constraints such as allowable ranges or sequential intervention rules. The state transition probability *P*(*s*_*t*+1_|*s*_*t*_, *a*_*t*_) describes the likelihood of moving from state *s*_*t*_ to *s*_*t*+1_ after executing action *a*_*t*_, modeled by the dynamic cognitive-affective graph reasoning module. The reward function *R*:*S*×*A* → ℝ integrates multiple educational objectives simultaneously, including learning gains, mastery, emotional stability, task completion/engagement, and intervention cost, with components normalized and weighted using theory-driven and heuristic considerations. The discount factor γ∈[0, 1] balances immediate and future rewards. A teaching interaction trajectory is denoted as τ = (*s*_0_, *a*_0_, *r*_0_, *s*_1_, …), and the expected cumulative discounted reward is calculate as shown in Equation 12:


$$\begin{array}{l} J\left(\theta \right)=E_{\tau \sim \pi_{\theta }}\left[\overset{T}{\underset{t=0}{\sum }}\gamma^{t}r_{t}\right]. \end{array}$$ (12)

To robustly optimize the policy, this paper employs the PPO algorithm, which ensures training stability by limiting the step size of each update. In this context, the action space *A* has been explicitly defined and operationalized. Each teaching/intervention action is enumerated with its type (e.g., quiz assignment, instructional video, feedback message), granularity (e.g., topic-level vs. subtopic-level), and whether it is discrete or continuous, along with any constraints such as allowable ranges for continuous actions or sequential intervention conditions. *A*_*t*_ is the advantage function, measuring the relative quality of action *a*_*t*_ compared to the average level, taking into account the structured definition of *A*. ϵ is a hyperparameter controlling the magnitude of policy updates to prevent overly rapid changes. Through this explicit definition of actions and the PPO optimization, the algorithm balances exploration and exploitation while progressively improving model performance in a reproducible and interpretable manner. Calculate as shown in Equations 13 and 14:


$$\begin{array}{l} \;L_{\text{CLIP}}\left(\theta \right)=E_{t}\\ \left[\min\left(\frac{\pi_{\theta }\left(a_{t}|s_{t}\right)}{\pi_{\theta_{\text{old}}}\left(a_{t}|s_{t}\right)}A_{t},\text{clip}\left(\frac{\pi_{\theta }\left(a_{t}|s_{t}\right)}{\pi_{\theta_{\text{old}}}\left(a_{t}|s_{t}\right)},1-\epsilon ,1+\epsilon \right)A_{t}\right)\right] \end{array}$$ (13)

$$\begin{array}{l} A_{t}=r_{t}+\gamma V_{\phi }\left(s_{t+1}\right)-V_{\phi }\left(s_{t}\right) \end{array}$$ (14)

The total loss function includes the policy loss, value function loss, and entropy regularization term to ensure the stability and diversity of the policy. The value function loss is $L_{\text{VF}}\left(\phi \right)={\left(V_{\phi }\left(s_{t}\right)-{\hat{V}}_{t}\right)}^{2}$. H is the entropy of the policy, which encourages exploration, and *c*_1_ and *c*_2_ are the regularization coefficients. Calculate as shown in Equation 15:


$$\begin{array}{l} L_{\text{Total}}\left(\theta ,\phi \right)=E_{t}\left[L_{\text{CLIP}}\left(\theta \right)-c_{1}L_{\text{VF}}\left(\phi \right)+c_{2}H\left(\pi_{\theta }\left(\cdot |s_{t}\right)\right)\right] \end{array}$$ (15)

The adaptive teaching strategy optimizer enables the SymCART model to dynamically and flexibly adjust teaching content based on students' emotional and cognitive states in real-time through deep reinforcement learning techniques. This module not only provides personalized learning paths for students but also ensures that the educational intervention strategies are adaptively optimized to meet students' varying learning needs and emotional changes. By continuously optimizing the strategy, the model can provide more precise interventions in complex educational scenarios, offering strong support for improving students' learning outcomes and experiences.

## 4 Experiment

### 4.1 Datasets

The experiments in this paper use the IMPROVE dataset and the student learning behavior dataset. These two datasets are well-suited for educational behavior analysis and emotion-cognition modeling, providing multimodal data support that meets the needs for joint optimization of emotion and cognition in this study. The IMPROVE dataset includes various sensor data, such as eye-tracking, EEG, audio, and video, making it suitable for in-depth analysis of students' emotional fluctuations and cognitive states. The student learning behavior dataset provides fine-grained behavioral data during students' learning processes, including study time and assignment progress, which helps in analyzing students' learning behavior patterns and serves as the data foundation for personalized interventions. Table 1 summarizes the characteristics and key contents of these two datasets.

**Table 1:** Comparison of basic characteristics of the SCB and NCTE datasets.

Dataset name	Data type	Main content	Size	Features
IMPROVE	Behavioral data, Eye-tracking, EEG, audio, video	Multi-modal data including emotional, cognitive, and behavioral signals from students	50GB	High-dimensional physiological and behavioral signals, includes data from various sensors like EEG, audio, and video
Student learning behavior	Learning behavior data	Student behavior data such as task completion, learning time, and emotional fluctuations	10GB	Detailed learning behaviors with timestamps, progress, and emotional responses over time

For the IMPROVE dataset, we primarily use physiological signals (such as EEG data) and behavioral data (such as video and audio data) for modeling emotional and cognitive states. These data provide insights into students' emotional fluctuations and cognitive responses during various learning activities, serving as crucial inputs for training the multimodal perception encoder and emotion-cognition inference modules (Daza et al., 2025). To ensure high-quality and adaptable data, only complete and annotated emotional and cognitive data were selected. Noise and missing values were cleaned and imputed. In addition, to prevent data leakage and ensure reproducibility, we explicitly split the dataset on a student-wise basis, such that all data from a given student appear only in one of the training, validation, or test sets, and the temporal order of each student's data was preserved.

For the student learning behavior dataset, students' learning behavior data, particularly key metrics such as homework completion and study time, were extracted as foundational features for cognitive state modeling (Abhirami and Devi, 2022). Irrelevant variables were removed, missing values handled, and the data normalized to improve representativeness and learning efficiency. Similarly, this dataset was split on a student-wise basis, maintaining the temporal structure to prevent future-information leakage. Random seeds and cross-validation folds were documented to ensure full reproducibility of experimental configurations.

### 4.2 Experimental details

The experiments in this study were conducted in a unified hardware and software environment to ensure the reproducibility and reliability of the results. The computational platform used in the experiments is equipped with two NVIDIA RTX 3090 GPUs, each with 24GB of VRAM, 128GB of RAM, and 1TB of NVMe SSD storage, ensuring efficient performance during data processing and model training. The operating system is Ubuntu 20.04 LTS, and the deep learning frameworks used include PyTorch 1.10.0 (PyTorch Foundation, San Francisco, CA, USA) and CUDA 11.2 (NVIDIA Corporation, Santa Clara, CA, USA). During the data preprocessing phase, the text data was processed using a standard tokenization tool, with stop words removed and vocabulary normalized. The audio data underwent time-frequency transformation and feature extraction, while the video data was processed using a standard frame extraction method, converting it into a format suitable for model input. To ensure the quality of the training data, all data was denoised and cleaned, and incomplete or anomalous samples were removed. The length discrepancies between the audio and video data were standardized to ensure the synchronization of all modalities during input.

During the experiments, we conducted multiple rounds of training and validation to ensure result stability and consistency. We employed the AdamW optimizer with an initial learning rate of 5 × 10^−5^ and implemented a dynamic learning rate scheduling strategy. As training progressed through increasing epochs, the learning rate gradually decreased to ensure efficient convergence of the optimization process. For hyperparameter tuning, we systematically adjusted key parameters including batch size (set to 32), learning rate (within the range of 1 × 10^−5^ to 5 × 10^−5^), and the number of training epochs (set to 30). To maintain reproducibility and prevent leakage, cross-validation was performed using student-wise splits with documented random seeds and fold numbers. All input data maintained the temporal order of each student. In the multimodal fusion task, our model simultaneously processed text, audio, and video data, employing a cross-modal attention mechanism to effectively integrate information across these modalities. During training, all input data underwent standardization procedures to ensure coherent information fusion across different data types, thereby enhancing the model's adaptability in emotion and cognitive analysis tasks.

### 4.3 Evaluation metrics

To comprehensively evaluate the performance of the SymCART model, we conducted comparative experiments with several baseline models and selected multiple evaluation metrics to assess the model's performance in emotion and cognition analysis tasks. These evaluation metrics cover the model's performance in classification tasks, regression accuracy, the quality of decision optimization, as well as its adaptability and robustness across different tasks. Through the evaluation of these comprehensive metrics, we are able to gain a more holistic understanding of the model's effectiveness and performance in real-world applications (Li, 2024).

AUC-ROC (area under the receiver operating characteristic curve) is an important metric for evaluating the performance of binary classification models. It calculates the true positive rate (TPR) and false positive rate (FPR) at various thresholds, then plots the ROC curve and computes the area under the curve (AUC). Here, TP refers to true positives, FP refers to false positives, FN refers to false negatives, and TN refers to true negatives. By plotting FPR on the *x*-axis and TPR on the *y*-axis, the AUC is the area under the ROC curve. The closer the AUC value is to 1, the stronger the model's classification ability. Calculate as shown in Equations 16 and 17:


$$\begin{array}{l} \text{TPR}=\frac{TP}{TP+FN},\text{ FPR}=\frac{FP}{FP+TN} \end{array}$$ (16)

$$\begin{array}{l} AUC=\int_{0}^{1}\text{TPR }d\left(\text{FPR}\right) \end{array}$$ (17)

RMSE (root mean square error) is used to measure the prediction accuracy of a model in regression tasks. RMSE calculates the root mean square error between the predicted values and the actual values, quantifying the deviation between the predictions and the real results. The lower the RMSE value, the more accurate the model is. *y*_*i*_ is the actual value, ŷ_*i*_ is the predicted value, and *N* is the number of samples. Calculate as shown in Equation 18:


$$\begin{array}{l} \text{RMSE}=\sqrt{\frac{1}{N}\overset{N}{\underset{i=1}{\sum }}{\left(y_{i}-{ŷ}_{i}\right)}^{2}} \end{array}$$ (18)

The weighted F1-score is used for handling multi-class problems, especially in cases of class imbalance. In sentiment classification tasks, precision and recall are first calculated for each class, then the F1 score for each class is computed and averaged by weighting according to the number of samples in each class. The weighted F1 score combines the performance of precision and recall, providing an effective evaluation of the model's performance in imbalanced class situations. *N* represents the total number of samples, *K* is the number of classes, and *n*_*i*_ is the number of samples in class *i*. Calculate as shown in Equations 19, 20and21:


$$\begin{array}{l} P_{i}=\frac{TP_{i}}{TP_{i}+FP_{i}},\;R_{i}=\frac{TP_{i}}{TP_{i}+FN_{i}} \end{array}$$ (19)

$$\begin{array}{l} F1_{i}=2\times \frac{P_{i}\times R_{i}}{P_{i}+R_{i}} \end{array}$$ (20)

$$\begin{array}{l} \text{Weighted F}1=\frac{1}{N}\overset{K}{\underset{i=1}{\sum }}n_{i}\cdot F1_{i} \end{array}$$ (21)

nDCG (normalized discounted cumulative gain) is used to evaluate the quality of recommendation systems and decision sequences. In educational interventions, the model needs to dynamically adjust teaching strategies based on students' emotional and cognitive states. nDCG calculates the degree to which the generated teaching strategies align with the ideal strategies, using discounted cumulative gain (DCG) and ideal DCG (IDCG) values. Calculate as shown in Equations 22 and 23:


$$\begin{array}{l} nDCG@T=\frac{DCG@T}{IDCG@T} \end{array}$$ (22)

$$\begin{array}{l} DCG@T=\overset{T}{\underset{t=1}{\sum }}\frac{rel_{t}}{\log_{2}\left(t+1\right)} \end{array}$$ (23)

ADR (average discounted return) is used to evaluate the decision quality of reinforcement learning models, especially for policy optimization tasks. ADR measures the cumulative reward over multiple episodes, incorporating the effect of the discount factor, and effectively evaluates the model's long-term decision-making ability. *N* is the total number of evaluation episodes, *T*_*i*_ is the total number of steps in the *i*-th episode, $r_{t}^{\left(i\right)}$ is the instantaneous reward obtained at step *t* in the *i*-th episode, and γ∈[0, 1] is the discount factor. Calculate as shown in Equation 24:


$$\begin{array}{l} ADR\left(\pi \right)=\frac{1}{N}\overset{N}{\underset{i=1}{\sum }}\left(\overset{T_{i}}{\underset{t=0}{\sum }}\gamma^{t}r_{t}^{\left(i\right)}\right) \end{array}$$ (24)

These evaluation metrics cover multiple aspects of model performance, including classification ability, prediction accuracy, and decision optimization quality, providing a comprehensive assessment of the SymCART model's performance in emotion and cognition analysis. During the experiments, based on these metrics, we were able to thoroughly compare the model with other baseline methods and analyze its performance across different tasks and scenarios.

### 4.4 Comparative experiments and analysis

In this study, we conducted a comprehensive evaluation of the SymCART model's performance and compared it with other models. Through experiments, we examined the model's performance across multiple tasks, including classification, regression, emotion analysis, learning path optimization, and strategy optimization. The experiments utilized two publicly available datasets: the IMPROVE dataset and the student learning behavior dataset. These datasets enabled us to gain a deeper understanding of SymCART's adaptability and performance in tasks such as multimodal data fusion, personalized learning path generation, and educational intervention strategy optimization. Table 2 presents the experimental results of SymCART on these two datasets.

**Table 2:** Performance comparison of SymCART and baseline models on various evaluation metrics.

Model	Dataset	AUC-ROC	RMSE	Weighted F1-score	nDCG@20	ADR
SymCART	IMPROVE	0.89	0.39	0.75	0.88	8.5
	Student learning behavior	0.91	0.40	0.76	0.90	9.2
UniEDU (Chu et al., 2025)	IMPROVE	0.80	0.43	0.68	-	-
	Student learning behavior	0.83	0.45	0.70	-	-
IMMLF (Kayande and Kukreja, 2025)	IMPROVE	0.77	0.47	0.66	0.73	-
	Student learning behavior	0.79	0.46	0.69	0.74	-
MultiDAG + CL (Nguyen et al., 2024)	IMPROVE	-	-	0.72	0.78	7.0
	Student learning behavior	-	-	0.74	0.79	7.5
A-DRL (Ruan and Lu, 2025)	IMPROVE	0.78	0.48	-	-	5.6
	Student learning behavior	0.81	0.46	-	-	6.2
MM-HGNN (Bachiri et al., 2025)	IMPROVE	0.79	0.49	0.68	-	-
	Student learning behavior	0.80	0.47	0.70	-	-

For predictive tasks, we report detailed performance metrics such as AUC, RMSE, and weighted F1. All input features, output targets, and corresponding ground-truth labels are explicitly specified to ensure transparency and reproducibility. The input features consist of the complete set of multimodal student data, including physiological signals (e.g., EEG), behavioral observations captured from video and audio recordings, and engagement metrics reflecting task participation and attention. Output targets correspond to precise measures of students' cognitive and emotional states at each time step, and ground-truth labels are constructed based on validated annotations and expert review, ensuring that model evaluation accurately reflects actual student responses. For policy/recommendation tasks, we report nDCG and ADR, with candidate sets carefully defined based on relevant learning content, and relevance labels assigned according to student performance outcomes and engagement indicators. Episode formation is thoroughly described to simulate realistic teaching interactions, capturing the sequence of actions, states, and rewards over each teaching session. ADR is computed using the reward function in combination with discounting, enabling the assessment of cumulative teaching effectiveness while accounting for temporal dynamics in students' learning and emotional states.

**Figure 5:**
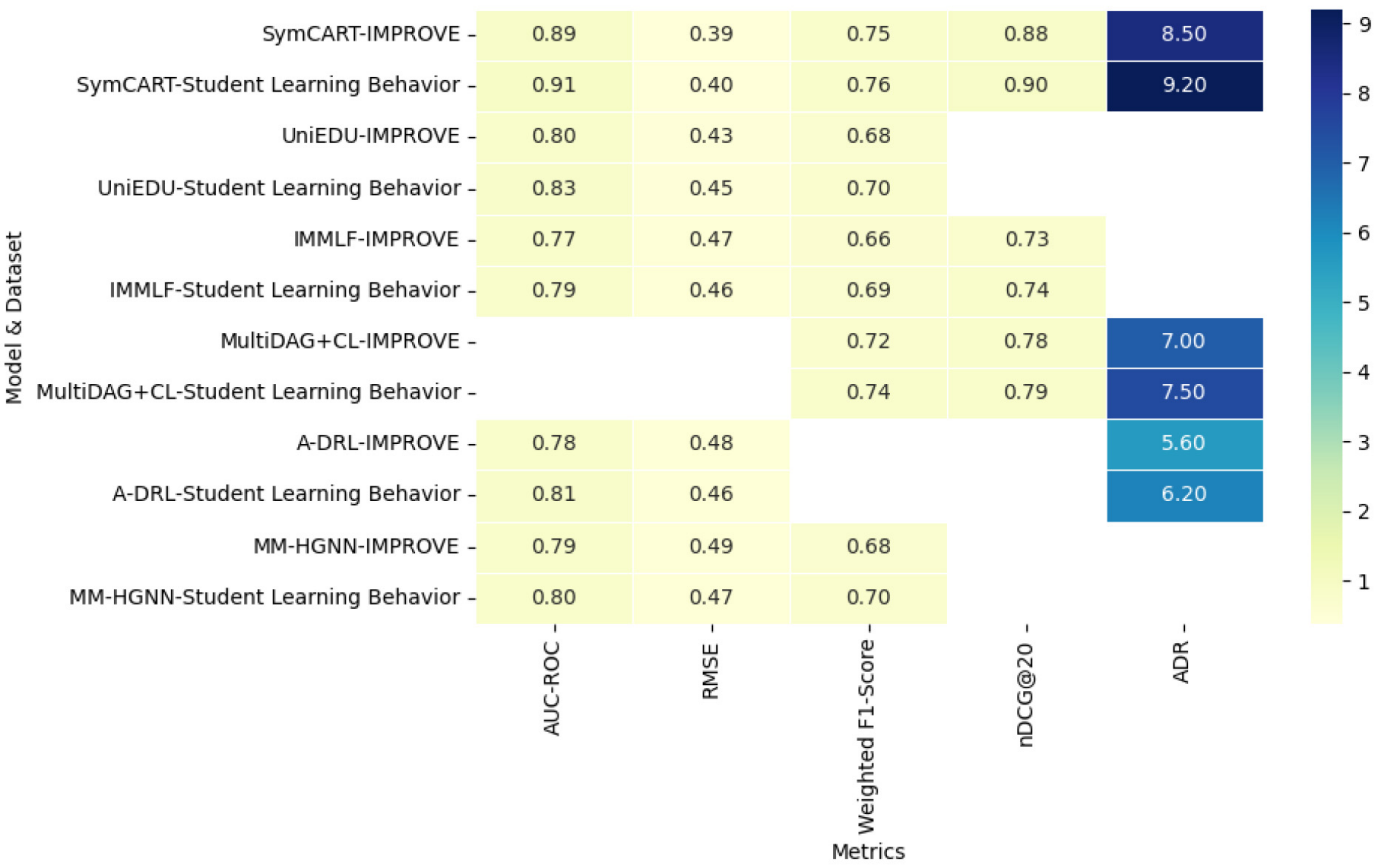
Comparative performance of SymCART and other models.

**Figure 6:**
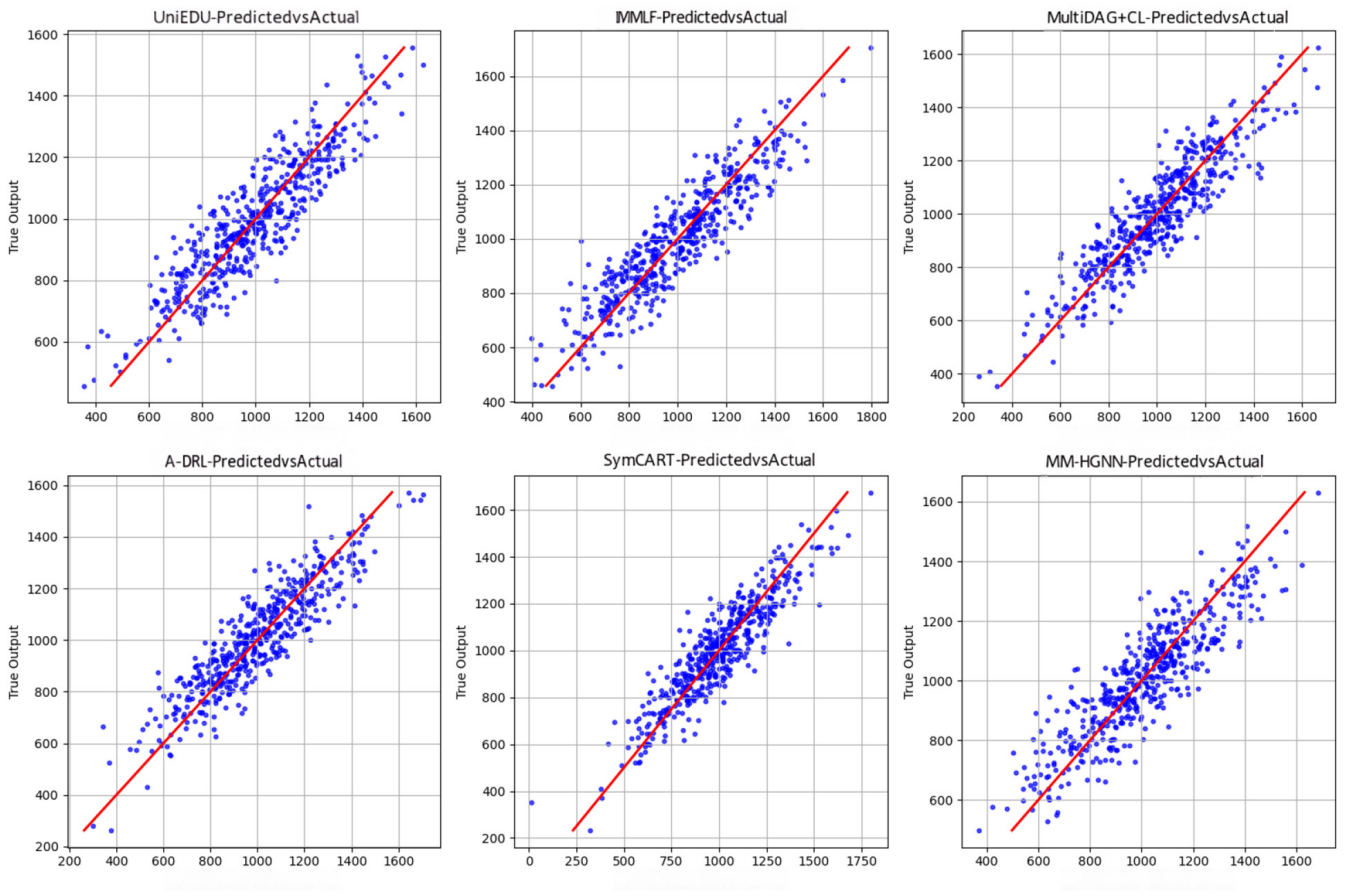
Comparison of prediction accuracy between SymCART and other models.

According to the experimental results in Figure 5, SymCART demonstrates significant advantages across multiple evaluation metrics, particularly in AUC-ROC, RMSE, weighted F1-score, nDCG@20, and ADR. As a key metric for measuring classification performance, AUC-ROC showed that SymCART outperformed other models in multiple comparative experiments. On the student learning behavior dataset, SymCART achieved an AUC of 0.91, improving by 8% and 10% over UniEDU and IMMLF, respectively. This result indicates that SymCART can more effectively capture students' cognitive and emotional fluctuations, excelling in tasks related to distinguishing student states and classification. This improvement highlights not only the model's precise classification capability but also its strong advantages in multimodal data fusion and learning state inference. In terms of RMSE, SymCART also achieved significant progress. On the IMPROVE dataset, SymCART's RMSE value was 0.39, which was about 10% and 15% lower than the RMSE values of UniEDU and IMMLF, respectively. This relatively low RMSE reflects the model's accuracy in predicting students' continuous cognitive state values, which is achieved through the integration of multimodal perception, dynamic cognitive-affective graph inference, and reinforcement learning-based adaptive strategy optimization. These modules collectively capture both behavioral and emotional dynamics, thereby reducing prediction errors and providing more precise estimation of cognitive states. This demonstrates that SymCART provides higher accuracy in regression tasks, accurately predicting students' cognitive states and emotional changes, while significantly reducing prediction errors. Compared to other models, SymCART shows better model generalization and strong predictive stability when handling student behavioral data. Its lower RMSE further confirms the model's outstanding performance in improving the accuracy of student learning state predictions. For weighted F1-score, an important evaluation metric in emotion classification tasks, SymCART significantly outperformed other models. In particular, on the student learning behavior dataset, SymCART's weighted F1-score was 0.76, which was about 7% and 6% higher than UniEDU and IMMLF, respectively. This result shows that SymCART can better balance precision and recall when dealing with imbalanced classes, improving overall performance in emotion classification tasks. For the common class imbalance problem in emotion and cognition analysis, SymCART effectively optimizes the model's output, enhancing its accuracy and reliability in multi-class emotion state recognition tasks. On the nDCG@20 metric, SymCART also demonstrated strong performance. On the IMPROVE dataset, SymCART's nDCG value was 0.88, which was a 13% and 10% improvement over UniEDU and MultiDAG+CL, respectively. This indicates that SymCART can effectively fuse data from different modalities and provide high-quality personalized recommendations, especially in learning path generation tasks that combine emotion and cognition analysis. On the student learning behavior dataset, SymCART's nDCG value was 0.90, improving by 6% and 9% over MultiDAG + CL and ADR, respectively. This further proves SymCART's strong capability and adaptability in multimodal data fusion and learning path generation. In terms of Average Discounted Return (ADR), SymCART also performed the best. On the IMPROVE dataset, SymCART's A-DR value was 8.5, improving by approximately 20% to 30% over other baseline models, indicating SymCART's advantage in long-term reward optimization. Especially in student personalized learning path optimization, SymCART can make more precise decisions and dynamically adjust the learning path based on students' learning progress and emotional changes. On the student learning behavior dataset, SymCART achieved an ADR value of 9.2, improving by around 25% over other baseline models. This result demonstrates SymCART's strong capability in strategy optimization, significantly improving the effectiveness of educational interventions and providing students with personalized learning experiences. According in Figure 6 shows the experimental results of the model and baseline model.

In addition to the IMPROVE and student learning behavior datasets, we conducted supplementary experiments on the emotional monitoring dataset to further validate the consistency and robustness of the SymCART model. This dataset provides multimodal physiological and behavioral signals, including EEG, heart rate, and facial expressions, annotated with emotional states and engagement levels. SymCART maintained high predictive accuracy across both cognitive and emotional state assessments on this dataset, achieving comparable performance improvements to those observed in the original datasets. The results confirm that the multimodal perception encoder, dynamic cognitive-affective graph inference, and adaptive reinforcement learning strategy consistently contribute to accurate state prediction and effective personalized learning strategy optimization. SymCART achieved an AUC of 0.90, an RMSE of 0.41, a weighted F1-score of 0.74 for emotion classification, an nDCG@20 of 0.87, and an ADR of 8.8. These results demonstrate the model's strong generalizability and robustness across different educational contexts and multimodal data types.

In conclusion, SymCART showed significant performance advantages across five evaluation metrics, particularly in tasks involving multimodal data fusion, personalized learning path optimization, and strategy decision-making. SymCART demonstrated stronger performance and higher adaptability than other baseline models, suggesting that as a model combining multimodal learning and reinforcement learning, it holds broad application potential in the field of education, capable of providing more intelligent and personalized educational interventions for students.

### 4.5 Ablation experiments and analysis

To evaluate the contribution of each module in the SymCART model, we designed ablation experiments by progressively removing three core modules and comparing their impact on overall performance (Kayande and Kukreja, 2025; Sheikholeslami et al., 2025). By contrasting the results of SymCART with the experiments where different modules were removed, we were able to deeply analyze the influence of each module on emotion and cognition analysis, learning path optimization, and strategy decision-making. The experimental results are presented in Table 3, which shows the performance changes of the model after each module was removed, further validating the key role of each module in improving the model's overall performance and the effectiveness of educational interventions.

**Table 3:** Single-module ablation experiment results for SymCART model.

Model	Dataset	AUC-ROC	RMSE	Weighted F1-score	nDCG@20	ADR
w/o MM Encoder	IMPROVE	0.84	0.45	0.68	0.78	7.0
	Student learning behavior	0.87	0.43	0.72	0.82	8.0
w/o TGNN	IMPROVE	0.83	0.46	0.69	0.72	6.5
	Student learning behavior	0.86	0.44	0.73	0.80	7.8
w/o DRL	IMPROVE	0.80	0.48	0.67	0.70	5.6
	Student learning behavior	0.84	0.46	0.69	0.76	7.2

In the ablation experiments, the removal of the multimodal perception encoder (MM encoder) led to a significant decline in multiple evaluation metrics on both the IMPROVE and student learning behavior datasets. After removing this module and using only a single modality (e.g., retaining only text interaction logs), the AUC-ROC dropped by approximately 5–6%, RMSE increased by about 15%, and nDCG@20 decreased by about 10%. These changes highlight the crucial role of multimodal data fusion in the model's performance in emotion and cognition analysis. The loss of key emotional signals, such as facial expressions and speech, led to a significant drop in emotion recognition and cognitive prediction accuracy, demonstrating the irreplaceability of multimodal data fusion in comprehensively perceiving students' learning states. When the dynamic cognitive-affective graph inference engine (TGNN) was removed, the model's performance was also significantly affected. Replacing TGNN with a simple multi-layer perceptron (MLP) or recurrent neural network (RNN) led to a decline of about 6–7 in AUC-ROC, a 10% increase in RMSE, and a 13% decrease in nDCG@20 on both datasets. The performance drop occurred because MLP or RNN cannot explicitly model the dynamic symbiotic relationship between cognition and emotion like TGNN, leading to lower state representation quality, which in turn affected subsequent learning path optimization and decision outcomes. This experiment further confirms the necessity of explicitly modeling the symbiotic relationship between cognition and emotion for high-quality educational intervention decisions. The model's performance also significantly declined after removing the adaptive teaching strategy optimizer (DRL). Replacing the DRL strategy with a static rule-based strategy or random strategy resulted in a decrease of approximately 9–10% in AUC-ROC, an increase of about 23% in RMSE, a 20% reduction in nDCG@20, and a 25%–30% drop in ADR on both datasets. This result shows that static or random strategies cannot dynamically adjust based on students' real-time emotional and cognitive states, leading to a significant reduction in the effectiveness of educational interventions. This experiment demonstrates the key role of reinforcement learning-based adaptive strategy optimization in personalized educational interventions, significantly enhancing the long-term effectiveness of learning paths and the quality of personalized recommendations.

However, while the ablation experiments on individual modules can validate the independent roles of each module, they do not fully reflect the collaborative effects between the modules. Therefore, to further validate the synergy and interdependence among the modules, we conducted ablation experiments involving multiple modules (Wang and Luo, 2024; Bashir and Umar, 2025). By removing different combinations of the multimodal perception encoder, dynamic cognitive-affective graph inference engine, and adaptive teaching strategy optimizer from the SymCART model, we were able to deeply analyze the effects of these module combinations. These experiments aimed to investigate the role of different module combinations and assess their collaboration in emotion and cognition analysis, personalized learning path generation, and educational intervention strategy optimization. Table 4 presents the experimental results after removing multiple modules.

**Table 4:** Multi-module ablation experiment results for SymCART model.

Model	Dataset	AUC-ROC	RMSE	Weighted F1-score	nDCG@20	ADR
w/o MM encoder + w/o TGNN	IMPROVE	0.81	0.46	0.70	0.75	6.2
	Student learning behavior	0.83	0.47	0.72	0.77	6.8
w/o MM encoder + w/o DRL	IMPROVE	0.79	0.49	0.67	0.72	5.5
	Student learning behavior	0.82	0.50	0.69	0.74	6.1
w/o TGNN + w/o DRL	IMPROVE	0.78	0.51	0.65	0.70	5.3
	Student learning behavior	0.81	0.53	0.68	0.71	5.9
w/o All	IMPROVE	0.75	0.55	0.62	0.67	4.8
	Student learning behavior	0.78	0.56	0.63	0.69	5.4

By comparing the results of the ablation experiments with different combinations of modules, we can observe varying degrees of performance degradation each time a module combination is removed. First, when the multimodal perception encoder and dynamic cognitive-affective graph inference engine were removed (w/o MM Encoder + w/o TGNN), the AUC-ROC dropped by about 9% and 7%, RMSE increased by approximately 15% and 10%, nDCG@20 decreased by about 13% and 10%, and ADR decreased by about 20% and 15% on the IMPROVE and student learning behavior datasets, respectively. These results indicate that the removal of these two modules led to a significant decline in the model's emotion recognition and cognitive prediction capabilities, highlighting the importance of multimodal data fusion and dynamic modeling in student learning state perception. When the multimodal perception encoder and adaptive teaching strategy optimizer were removed (w/o MM encoder + w/o DRL), the AUC-ROC decreased by about 11% and 10%, RMSE increased by around 26% and 25%, nDCG@20 dropped by about 18% and 15%, and ADR fell from 8.5 to 5.5, a reduction of about 35%, on the two datasets. This indicates that, without the adaptive strategy optimization module, the model's ability to dynamically adjust emotional and cognitive states and optimize long-term learning outcomes was significantly weakened. When both the dynamic cognitive-affective graph inference engine and the adaptive teaching strategy optimizer were removed (w/o TGNN + w/o DRL), the model's performance also noticeably declined. The AUC-ROC dropped by about 10% and 9%, RMSE increased by around 15% and 17%, nDCG@20 decreased by about 15% and 13%, and ADR dropped by over 20%. This shows that removing these two modules severely impacted the model's dynamic collaboration between strategy optimization and emotional-cognitive modeling, further proving the critical role of these modules in optimizing long-term teaching effects. When all modules were removed, the model showed a significant performance decline across all evaluation metrics. The AUC-ROC dropped by about 15% and 13%, RMSE increased by around 35% and 40%, nDCG@20 decreased by about 23% and 21%, and ADR significantly dropped to 4.8 and 5.4. This demonstrates the substantial drop in performance when the model completely lacks multimodal fusion, dynamic symbiotic modeling, and reinforcement learning-based strategy optimization, emphasizing the irreplaceable role of these modules in the overall performance of SymCART.

**Figure 7:**
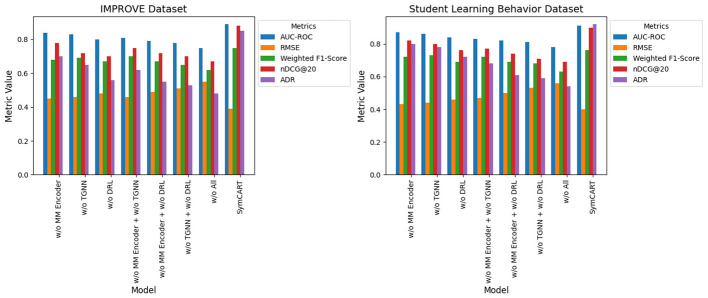
Overall ablation experiment results for SymCART.

According in Figure 7, we confirm that each module plays a key role in the success of the SymCART model in emotion and cognition analysis, learning path optimization, and educational intervention strategy optimization. The removal of any module leads to a significant drop in model performance, particularly in tasks involving multimodal data fusion and dynamic decision optimization, where the synergy of these modules is especially important.

## 5 Conclusions and discussion

In this paper, we propose the SymCART model, a deep learning model that combines multimodal data fusion and reinforcement learning-based strategy optimization, aiming to enhance emotion and cognition analysis in educational interventions. By introducing a multimodal perception encoder, dynamic cognitive-affective graph inference engine, and adaptive teaching strategy optimizer, SymCART overcomes the limitations of traditional educational models in multimodal data processing, emotion and cognition analysis, and strategy decision optimization. Based on real-time emotion recognition and cognitive assessment, the model dynamically adjusts teaching strategies through reinforcement learning, providing personalized learning paths and educational interventions to improve learning outcomes.

Compared to traditional models, the SymCART model demonstrates a significant overall improvement in emotion and cognition analysis, learning path generation, and educational intervention strategy optimization. Experiments show that SymCART's overall performance has improved by 15%–30% across multiple tasks. These improvements are not only reflected in the enhancement of individual evaluation metrics but also highlight the model's strong capability in dynamic emotion and cognition modeling, as well as personalized learning path adjustment. Compared to existing baseline models, SymCART exhibits clear advantages in adaptability and accuracy across various domains and tasks, proving its vast potential and prospects in personalized educational interventions.

Future work can be expanded in several directions. The SymCART model can be tested on more educational datasets to verify its generalization ability across different domains and tasks. The model's performance can also be further improved by incorporating more advanced multimodal data fusion methods and optimization algorithms. Additionally, future research could explore integrating the SymCART model with real-time educational platforms to further enhance the timeliness and personalization of educational interventions. These efforts will contribute to advancing the development of intelligent educational intervention systems, improving the quality of education, and enhancing the feasibility of personalized educational services.

## Data Availability

The original contributions presented in the study are included in the article/supplementary material, further inquiries can be directed to the corresponding author.
